# 1-[3-(2-Nitro­phen­yl)-5-phenyl-2-pyrazolin-1-yl]ethanone

**DOI:** 10.1107/S1600536810020611

**Published:** 2010-06-05

**Authors:** Huan-Mei Guo, Ben-Yu Huang, Xiang Qin, Huan-Ze Zou, Fang-Fang Jian

**Affiliations:** a Microscale Science Institute, Weifang University, Weifang 261061, People’s Republic of China; b Department of Chemistry & Chemical Engineering, Weifang University, Weifang 261061, People’s Republic of China

## Abstract

The title compound, C_17_H_15_N_3_O_3_, was prepared from 1-(2-nitro­phen­yl)-3-phenyl­prop-2-en-1-one and hydrazine. The dihedral angle between the benzene and phenyl rings is 74.55 (2)°. The pyrazoline ring is in a slight envelope conformation with the C atom bonded to the phenyl ring forming the flap. In the crystal structure, weak inter­molecular C—H⋯O hydrogen bonds connect mol­ecules into chains along [100].

## Related literature

For the biological activity of pyrazoline and its derivatives, see: Rawal *et al.* (1963 ▶); Dhal *et al.* (1975 ▶); Lombardino & Ottemes (1981 ▶); Manna *et al.* (2002 ▶). For related structures, see: Guo *et al.* (2006 ▶); Fahrni *et al.* (2003 ▶); Kimura *et al.* (1977 ▶).
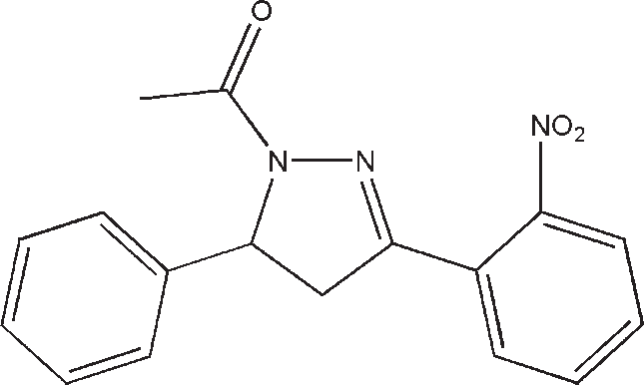


## Experimental

### 

#### Crystal data

C_17_H_15_N_3_O_3_
*M* *_r_* = 309.32
Monoclinic, 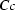

*a* = 6.5064 (13) Å
*b* = 12.385 (3) Å
*c* = 18.752 (4) Å
β = 98.26 (3)°
*V* = 1495.4 (5) Å^3^
*Z* = 4
Mo *K*α radiation
μ = 0.10 mm^−1^
*T* = 293 K
0.22 × 0.20 × 0.18 mm

#### Data collection

Bruker SMART CCD diffractometer
7203 measured reflections
1710 independent reflections
1354 reflections with *I* > 2σ(*I*)
*R*_int_ = 0.024

#### Refinement

*R*[*F* ^2^ > 2σ(*F* ^2^)] = 0.030
*wR*(*F* ^2^) = 0.087
*S* = 1.13
1710 reflections
208 parameters
2 restraints
H-atom parameters constrained
Δρ_max_ = 0.20 e Å^−3^
Δρ_min_ = −0.13 e Å^−3^

### 

Data collection: *SMART* (Bruker, 1997 ▶); cell refinement: *SAINT* (Bruker, 1997 ▶); data reduction: *SAINT*; program(s) used to solve structure: *SHELXS97* (Sheldrick, 2008 ▶); program(s) used to refine structure: *SHELXL97* (Sheldrick, 2008 ▶); molecular graphics: *SHELXTL* (Sheldrick, 2008 ▶); software used to prepare material for publication: *SHELXTL*.

## Supplementary Material

Crystal structure: contains datablocks global, I. DOI: 10.1107/S1600536810020611/lh5050sup1.cif

Structure factors: contains datablocks I. DOI: 10.1107/S1600536810020611/lh5050Isup2.hkl

Additional supplementary materials:  crystallographic information; 3D view; checkCIF report

## Figures and Tables

**Table 1:** Hydrogen-bond geometry (Å, °)

*D*—H⋯*A*	*D*—H	H⋯*A*	*D*⋯*A*	*D*—H⋯*A*
C6—H6*A*⋯O3^i^	0.93	2.41	3.293 (4)	157

Symmetry code: (i) 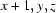.

**Fig. 1.**
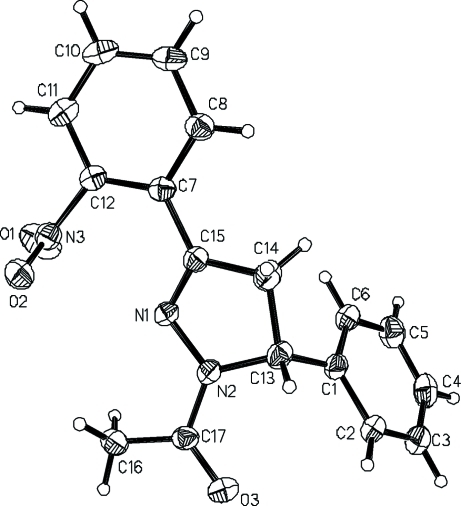
The molecular structure of the title compound with the atom-labeling scheme. Displacement ellipsoids are drawn at the 30% probability level.

## supplementary crystallographic information

### Comment

Pyrazoline and its derivatives are important and useful five-membered heterocyclic compounds, which are found to possess antiviral (Rawal *et al.*, 1963), antifungal (Dhal *et al.*,1975) and immunosuppressive activities (Lombardino & Ottemes, 1981). 1-Acetyl-3,5-diaryl-2-pyrazolines have been found to inhibit monoamine oxidases (Manna *et al.*, 2002). As part of our ongoing investigation of pyrazolines and their metal complexes, we report herein the crystal structure of the title compound, (I).

In the structure of (I) (Fig. 1), the bond lengths and angles are comparable with those in related structures (Guo *et al.*,2006; Fahrni *et al.*, 2003; Kimura *et al.*, 1977). The dihehral angle between the benzene and phenyl rings is 74.55 (2)°. The pyrazoline ring is in a slight envelope conformation with atom C13 deviating by 0.158 (4) Å form the essentially planar atoms N1/N2/C14/C15 (rms deviation = 0.003 Å). In the crystal structure, weak intermolecular C—H···O hydrogen bonds connect molecules into chains along [100].

### Experimental

3-phenyl-1-(2-nitrophenyl)-2-propen-1-one (0.01 mol) and hydrazine (0.03 mol, 80%) were mixed in acetic acid (30 ml) and stirred under reflux for 6 h; the mixture was then poured into ice-water to afford colourless solids. The solids were filtered off and washed with water until the pH of the solution was about 7.0. Finally, the crystals were dried at room temperature. Single crystals of compound (I) suitable for X-ray measurements were obtained by recrystallization from EtOH at room temperature.

### Refinement

In the absence of anomalous dispersion effects the Freidel pairs were merged. All H atoms were fixed geometrically and allowed to ride on their attached atoms, with C—H distances in the range 0.93–0.98Å and with *U*_iso_=1.2–1.5Ueq.

### Crystal data

**Table:**

C_17_H_15_N_3_O_3_	*F*(000) = 648
*M**_r_* = 309.32	*D*_x_ = 1.374 Mg m^−^^3^
Monoclinic, *C**c*	Mo *K*α radiation, λ = 0.71073 Å
Hall symbol: C -2yc	Cell parameters from 1354 reflections
*a* = 6.5064 (13) Å	θ = 3.3–27.3°
*b* = 12.385 (3) Å	µ = 0.10 mm^−^^1^
*c* = 18.752 (4) Å	*T* = 293 K
β = 98.26 (3)°	Bar, colourless
*V* = 1495.4 (5) Å^3^	0.22 × 0.20 × 0.18 mm
*Z* = 4	

### Data collection

**Table:**

Bruker SMART CCD diffractometer	1354 reflections with *I* > 2σ(*I*)
Radiation source: fine-focus sealed tube	*R*_int_ = 0.024
graphite	θ_max_ = 27.5°, θ_min_ = 3.3°
φ and ω scans	*h* = −8→7
7203 measured reflections	*k* = −16→16
1710 independent reflections	*l* = −24→24

### Refinement

**Table:**

Refinement on *F*^2^	Primary atom site location: structure-invariant direct methods
Least-squares matrix: full	Secondary atom site location: difference Fourier map
*R*[*F*^2^ > 2σ(*F*^2^)] = 0.030	Hydrogen site location: inferred from neighbouring sites
*wR*(*F*^2^) = 0.087	H-atom parameters constrained
*S* = 1.13	*w* = 1/[σ^2^(*F*_o_^2^) + (0.0483*P*)^2^ + 0.1147*P*] where *P* = (*F*_o_^2^ + 2*F*_c_^2^)/3
1710 reflections	(Δ/σ)_max_ < 0.001
208 parameters	Δρ_max_ = 0.20 e Å^−^^3^
2 restraints	Δρ_min_ = −0.13 e Å^−^^3^

### Special details

**Table:**

Geometry. All e.s.d.'s (except the e.s.d. in the dihedral angle between two l.s. planes) are estimated using the full covariance matrix. The cell e.s.d.'s are taken into account individually in the estimation of e.s.d.'s in distances, angles and torsion angles; correlations between e.s.d.'s in cell parameters are only used when they are defined by crystal symmetry. An approximate (isotropic) treatment of cell e.s.d.'s is used for estimating e.s.d.'s involving l.s. planes.
Refinement. Refinement of *F*^2^ against ALL reflections. The weighted *R*-factor *wR* and goodness of fit *S* are based on *F*^2^, conventional *R*-factors *R* are based on *F*, with *F* set to zero for negative *F*^2^. The threshold expression of *F*^2^ > σ(*F*^2^) is used only for calculating *R*-factors(gt) *etc*. and is not relevant to the choice of reflections for refinement. *R*-factors based on *F*^2^ are statistically about twice as large as those based on *F*, and *R*- factors based on ALL data will be even larger.

### Fractional atomic coordinates and isotropic or equivalent isotropic displacement parameters (Å^2^)

**Table:**

	*x*	*y*	*z*	*U*_iso_*/*U*_eq_	
C7	0.1900 (3)	0.67920 (17)	0.28552 (12)	0.0384 (5)	
N1	−0.1077 (3)	0.79477 (16)	0.27976 (10)	0.0373 (4)	
C12	0.2478 (3)	0.68202 (17)	0.36067 (13)	0.0381 (5)	
N2	−0.2744 (3)	0.82918 (16)	0.23108 (10)	0.0410 (4)	
O3	−0.5900 (3)	0.90449 (18)	0.20896 (11)	0.0596 (5)	
C15	0.0046 (3)	0.73132 (18)	0.24741 (12)	0.0376 (5)	
C8	0.3234 (4)	0.6223 (2)	0.24684 (14)	0.0455 (5)	
H8A	0.2919	0.6180	0.1970	0.055*	
N3	0.1191 (3)	0.73560 (17)	0.40852 (11)	0.0455 (5)	
O2	−0.0409 (3)	0.69121 (17)	0.41903 (11)	0.0593 (5)	
C17	−0.4397 (4)	0.8805 (2)	0.25303 (13)	0.0408 (5)	
C16	−0.4249 (4)	0.9042 (2)	0.33180 (14)	0.0485 (6)	
H16A	−0.5493	0.9399	0.3411	0.073*	
H16B	−0.3075	0.9502	0.3464	0.073*	
H16C	−0.4083	0.8379	0.3585	0.073*	
C9	0.5016 (4)	0.5720 (2)	0.28093 (17)	0.0520 (6)	
H9A	0.5873	0.5345	0.2539	0.062*	
C13	−0.2630 (4)	0.7933 (2)	0.15621 (12)	0.0438 (5)	
H13A	−0.3902	0.7543	0.1372	0.053*	
C1	−0.2353 (4)	0.8890 (2)	0.10825 (11)	0.0427 (5)	
C2	−0.4070 (4)	0.9335 (2)	0.06620 (13)	0.0505 (6)	
H2A	−0.5363	0.9012	0.0652	0.061*	
O1	0.1846 (4)	0.8190 (2)	0.43697 (15)	0.0842 (8)	
C11	0.4254 (4)	0.6332 (2)	0.39490 (15)	0.0483 (6)	
H11A	0.4595	0.6379	0.4447	0.058*	
C10	0.5517 (4)	0.5776 (2)	0.35494 (18)	0.0557 (7)	
H10A	0.6709	0.5437	0.3777	0.067*	
C14	−0.0785 (5)	0.7133 (2)	0.16914 (13)	0.0533 (6)	
H14A	−0.1249	0.6394	0.1605	0.064*	
H14B	0.0254	0.7300	0.1385	0.064*	
C6	−0.0422 (4)	0.9367 (2)	0.10715 (13)	0.0503 (6)	
H6A	0.0754	0.9071	0.1342	0.060*	
C5	−0.0242 (5)	1.0285 (3)	0.06587 (15)	0.0595 (7)	
H5A	0.1052	1.0603	0.0657	0.071*	
C3	−0.3884 (5)	1.0259 (3)	0.02551 (14)	0.0614 (7)	
H3A	−0.5054	1.0560	−0.0015	0.074*	
C4	−0.1966 (5)	1.0726 (3)	0.02533 (14)	0.0633 (8)	
H4A	−0.1836	1.1340	−0.0022	0.076*	

### Atomic displacement parameters (Å^2^)

**Table:**

	*U*^11^	*U*^22^	*U*^33^	*U*^12^	*U*^13^	*U*^23^
C7	0.0380 (11)	0.0305 (11)	0.0467 (12)	0.0000 (9)	0.0063 (10)	0.0020 (9)
N1	0.0378 (10)	0.0350 (10)	0.0380 (9)	0.0039 (8)	0.0018 (8)	0.0045 (7)
C12	0.0385 (12)	0.0295 (11)	0.0452 (11)	−0.0034 (9)	0.0027 (10)	−0.0010 (9)
N2	0.0412 (10)	0.0440 (11)	0.0367 (9)	0.0071 (9)	0.0013 (8)	0.0042 (8)
O3	0.0403 (10)	0.0773 (14)	0.0593 (10)	0.0098 (9)	0.0003 (9)	0.0081 (9)
C15	0.0407 (12)	0.0329 (12)	0.0389 (11)	0.0003 (9)	0.0044 (10)	0.0005 (9)
C8	0.0484 (13)	0.0356 (13)	0.0532 (13)	0.0031 (10)	0.0097 (11)	0.0009 (10)
N3	0.0442 (11)	0.0482 (12)	0.0426 (10)	−0.0021 (10)	0.0008 (9)	−0.0013 (9)
O2	0.0479 (10)	0.0729 (13)	0.0590 (11)	−0.0043 (9)	0.0140 (9)	0.0091 (9)
C17	0.0342 (11)	0.0398 (13)	0.0484 (12)	−0.0009 (9)	0.0063 (10)	0.0061 (10)
C16	0.0465 (13)	0.0472 (14)	0.0542 (14)	0.0025 (11)	0.0153 (11)	0.0020 (12)
C9	0.0420 (13)	0.0403 (14)	0.0751 (18)	0.0055 (11)	0.0128 (13)	−0.0027 (12)
C13	0.0474 (14)	0.0434 (13)	0.0381 (12)	0.0024 (10)	−0.0030 (10)	−0.0042 (10)
C1	0.0454 (13)	0.0489 (14)	0.0321 (10)	0.0046 (10)	−0.0006 (10)	−0.0062 (9)
C2	0.0465 (13)	0.0651 (17)	0.0378 (11)	0.0047 (12)	−0.0015 (10)	0.0027 (11)
O1	0.0747 (14)	0.0793 (16)	0.1018 (18)	−0.0201 (12)	0.0239 (13)	−0.0516 (14)
C11	0.0477 (14)	0.0379 (13)	0.0553 (14)	−0.0013 (11)	−0.0065 (11)	0.0005 (11)
C10	0.0402 (13)	0.0409 (14)	0.0829 (19)	0.0066 (12)	−0.0018 (13)	0.0042 (13)
C14	0.0695 (17)	0.0472 (15)	0.0409 (12)	0.0148 (13)	−0.0004 (12)	−0.0066 (10)
C6	0.0459 (14)	0.0654 (17)	0.0372 (12)	0.0006 (13)	−0.0026 (10)	−0.0036 (11)
C5	0.0654 (17)	0.0661 (18)	0.0475 (13)	−0.0154 (15)	0.0103 (13)	−0.0063 (13)
C3	0.0671 (18)	0.074 (2)	0.0427 (13)	0.0159 (16)	0.0055 (12)	0.0105 (13)
C4	0.086 (2)	0.0612 (19)	0.0443 (14)	−0.0036 (16)	0.0155 (15)	0.0074 (12)

### Geometric parameters (Å, °)

**Table:**

C7—C8	1.399 (3)	C9—H9A	0.9300
C7—C12	1.406 (3)	C13—C1	1.514 (4)
C7—C15	1.461 (3)	C13—C14	1.548 (4)
N1—C15	1.283 (3)	C13—H13A	0.9800
N1—N2	1.381 (3)	C1—C2	1.386 (3)
C12—C11	1.378 (3)	C1—C6	1.392 (4)
C12—N3	1.471 (3)	C2—C3	1.390 (4)
N2—C17	1.363 (3)	C2—H2A	0.9300
N2—C13	1.484 (3)	C11—C10	1.375 (4)
O3—C17	1.223 (3)	C11—H11A	0.9300
C15—C14	1.506 (3)	C10—H10A	0.9300
C8—C9	1.388 (4)	C14—H14A	0.9700
C8—H8A	0.9300	C14—H14B	0.9700
N3—O1	1.212 (3)	C6—C5	1.390 (4)
N3—O2	1.218 (3)	C6—H6A	0.9300
C17—C16	1.496 (3)	C5—C4	1.374 (4)
C16—H16A	0.9600	C5—H5A	0.9300
C16—H16B	0.9600	C3—C4	1.376 (5)
C16—H16C	0.9600	C3—H3A	0.9300
C9—C10	1.381 (4)	C4—H4A	0.9300
			
C8—C7—C12	115.8 (2)	N2—C13—H13A	109.7
C8—C7—C15	120.0 (2)	C1—C13—H13A	109.7
C12—C7—C15	124.2 (2)	C14—C13—H13A	109.7
C15—N1—N2	108.84 (18)	C2—C1—C6	118.6 (2)
C11—C12—C7	122.8 (2)	C2—C1—C13	119.7 (2)
C11—C12—N3	115.2 (2)	C6—C1—C13	121.7 (2)
C7—C12—N3	121.9 (2)	C1—C2—C3	120.9 (3)
C17—N2—N1	121.55 (18)	C1—C2—H2A	119.5
C17—N2—C13	125.0 (2)	C3—C2—H2A	119.5
N1—N2—C13	113.05 (17)	C10—C11—C12	119.5 (2)
N1—C15—C7	121.70 (19)	C10—C11—H11A	120.2
N1—C15—C14	113.7 (2)	C12—C11—H11A	120.2
C7—C15—C14	124.5 (2)	C11—C10—C9	120.0 (2)
C9—C8—C7	121.8 (3)	C11—C10—H10A	120.0
C9—C8—H8A	119.1	C9—C10—H10A	120.0
C7—C8—H8A	119.1	C15—C14—C13	102.69 (19)
O1—N3—O2	124.6 (2)	C15—C14—H14A	111.2
O1—N3—C12	117.3 (2)	C13—C14—H14A	111.2
O2—N3—C12	118.0 (2)	C15—C14—H14B	111.2
O3—C17—N2	119.9 (2)	C13—C14—H14B	111.2
O3—C17—C16	123.5 (2)	H14A—C14—H14B	109.1
N2—C17—C16	116.6 (2)	C5—C6—C1	120.3 (2)
C17—C16—H16A	109.5	C5—C6—H6A	119.9
C17—C16—H16B	109.5	C1—C6—H6A	119.9
H16A—C16—H16B	109.5	C4—C5—C6	120.4 (3)
C17—C16—H16C	109.5	C4—C5—H5A	119.8
H16A—C16—H16C	109.5	C6—C5—H5A	119.8
H16B—C16—H16C	109.5	C4—C3—C2	119.9 (3)
C10—C9—C8	120.1 (2)	C4—C3—H3A	120.1
C10—C9—H9A	120.0	C2—C3—H3A	120.1
C8—C9—H9A	120.0	C5—C4—C3	120.0 (3)
N2—C13—C1	110.74 (19)	C5—C4—H4A	120.0
N2—C13—C14	100.74 (17)	C3—C4—H4A	120.0
C1—C13—C14	116.0 (2)		

### Hydrogen-bond geometry (Å, °)

**Table:**

*D*—H···*A*	*D*—H	H···*A*	*D*···*A*	*D*—H···*A*
C6—H6A···O3^i^	0.93	2.41	3.293 (4)	157

Symmetry codes: (i) *x*+1, *y*, *z*.
